# Use of Intravenous Tranexamic Acid for Acute Management of Active Bleeding From Iris Microhaemangioma Presenting as Spontaneous Hyphema

**DOI:** 10.7759/cureus.90014

**Published:** 2025-08-13

**Authors:** Umar Ahmed, Elizabeth Saldana, Matthew Seager, Tasnim Kamal

**Affiliations:** 1 Ophthalmology, East Sussex Healthcare National Health Service (NHS) Trust, Eastbourne, GBR; 2 Ophthalmology, University of Buckinghamshire Medical School, Buckingham, GBR; 3 Plastic Surgery, Queen Victoria Hospital National Health Service (NHS) Foundation Trust, London, GBR

**Keywords:** anterior segment, eye emergency, iris microhaemangioma, ophthalmology, spontaneous hyphema

## Abstract

Iris microhaemangiomas, or Cobb’s tufts, are rare, benign vascular lesions that may cause spontaneous hyphema and elevated intraocular pressure, risking vision loss. Due to the rarity of these cases, there is no consensus on acute management. We present a case of a woman in her seventies who presented with right eye pain and blurred vision due to spontaneous hyphema secondary to active bleeding from a vascular tuft. Conservative measures, including mydriatics and compression, failed to control the haemorrhage. After multidisciplinary consultation, 1 gram of intravenous tranexamic acid was administered, achieving haemostasis within 30 minutes without adverse effects. Imaging confirmed bilateral iris microhaemangiomas. Over six months of follow-up, the patient’s vision and intraocular pressures remained stable with no recurrence. This case suggests intravenous tranexamic acid may be a low-risk and effective option for managing spontaneous hyphema secondary to iris microhaemangiomas when initial treatments are unsuccessful.

## Introduction

Iris microhaemangiomas, also known as Cobb’s tufts, are rare, benign, acquired vascular lesions of the iris characterised by tightly coiled blood vessels at the pupillary margin. Age at presentation ranges from the forties to the eighties, with no racial or sexual associations. They are usually bilateral and associated with systemic conditions such as diabetes, myotonic dystrophy, and various cardiorespiratory disorders [1]. Typically asymptomatic, they occasionally cause spontaneous hyphema, leading to elevated intraocular pressures (IOPs) and corneal blood staining, risking vision loss [2]. With no consensus on managing these spontaneous bleeds, this case demonstrates rapid haemostasis using intravenous tranexamic acid.

## Case presentation

A woman in her seventies presented to the ophthalmology emergency clinic with a four-day history of right eye pain and progressive blurred vision. She denied trauma, medication changes, or precipitating events. Notably, she had a similar episode of spontaneous hyphema of unknown cause in the same eye five years earlier, with an associated blood pressure (BP) of 216/108. Her medical history included hypertension (managed with amlodipine 5 mg), white coat syndrome, and prediabetes. She had no history of anticoagulant use.

On examination, best-corrected visual acuity was 6/9 in the right eye (previously 6/6) and 6/7.5 in the left. IOPs were 14 mmHg and 11 mmHg, respectively. Slit-lamp examination of the right eye revealed a stream of active bleeding from a tightly coiled vascular tuft at the 11 o’clock position, a 4 mm hyphema, and early corneal blood staining (Video 1). A similar lesion was suspected in the left eye. There was no evidence of iris neovascularisation with open angles on gonioscopy. Fundoscopy showed no retinal vascular disease, and a clear vitreous. On arrival, the patient had a systolic BP of >200mmHg, which normalised under observation.

**Video 1:** Right eye active bleeding from iris vascular tuft at 11 o'clock

**Figure 1:**
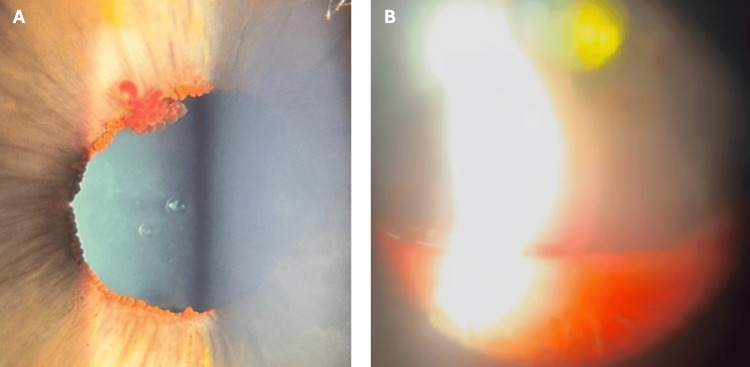
Anterior segment imaging once bleeding stopped A: Right eye iris vascular tuft at 11 o'clock, once haemostasis is achieved B: Right eye 4 mm residual hyphema once haemostasis is achieved

Initial management with compression via a three-mirror lens and mydriatic agents (phenylephrine 2.5% and tropicamide 1%) was unsuccessful. After two hours of persistent bleeding, multidisciplinary consultation was sought; however, there was no clear consensus on further management. It was decided to administer 1 g intravenous tranexamic acid based on the experience of safe use in oculoplastic theatre cases and after discussions with a consultant anaesthetist. Haemostasis was achieved within 30 minutes after infusion (Figure 1A, 1B). The patient was discharged with topical dexamethasone 0.1% QDS and dorzolamide 2% BD. Follow-up at days one and four confirmed complete resolution of hyphema. No adverse effects from tranexamic acid were observed.

**Figure 2:**
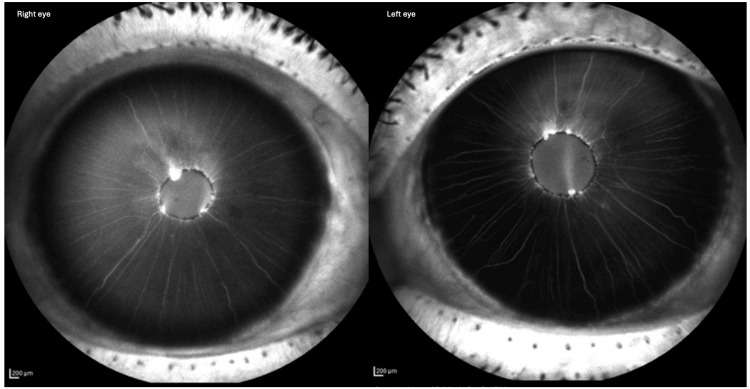
Iris fluorescein angiography Iris fluorescein angiography of both eyes showing multiple iris microhaemangiomas on the pupillary margin of both eyes with leak

**Figure 3:**
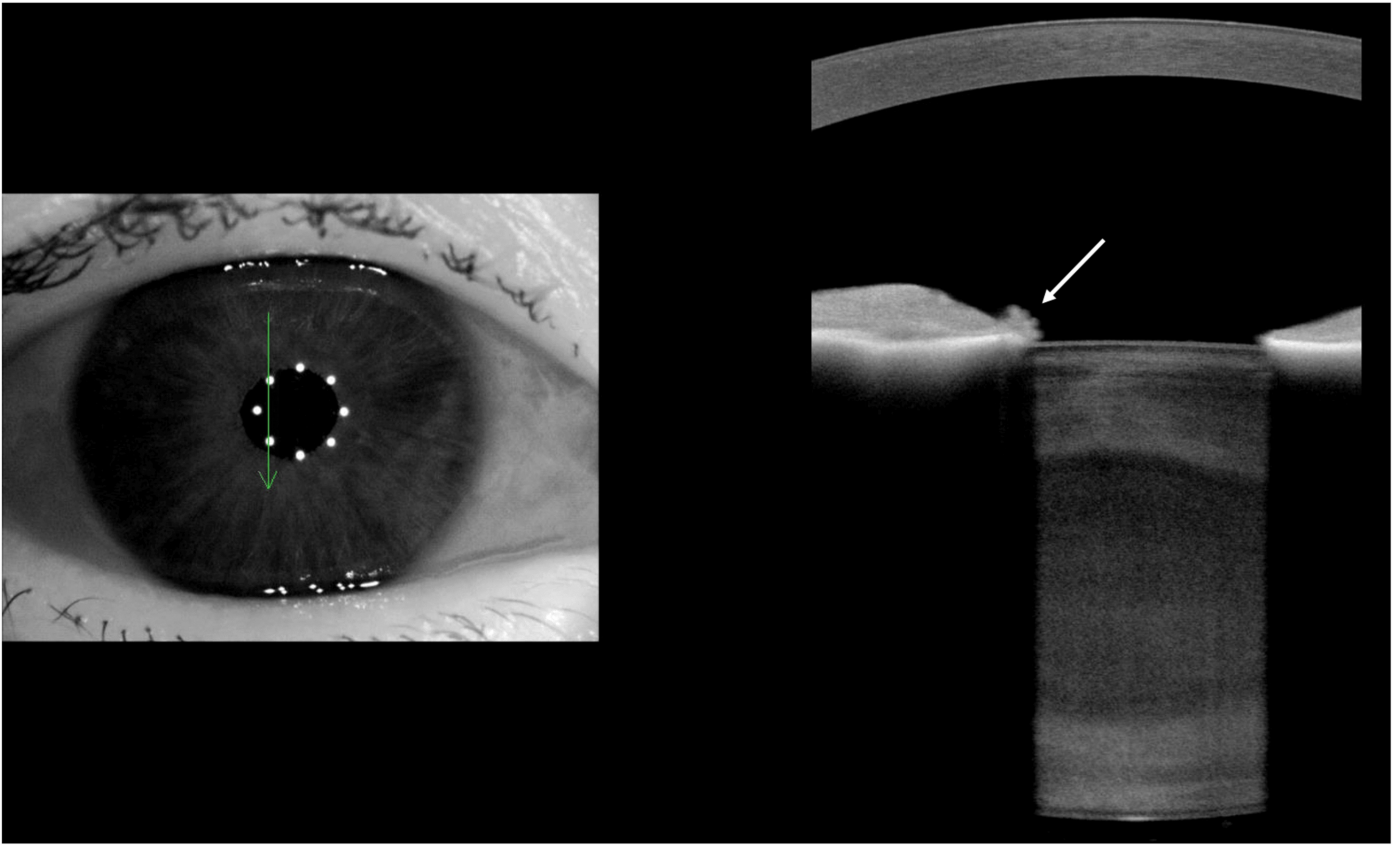
Right eye iris anterior segment OCT Right eye iris anterior segment optical coherence tomography (OCT) demonstrating nodular thickening corresponding to the bleeding iris microhaemangioma shown by white arrow

Differential diagnoses included iris melanoma and arteriovenous malformation. Routine blood tests (full blood count (FBC), urea and electrolytes (U&E), coagulation screen, random blood glucose and glycated haemoglobin (HbA1C)) were normal. Optical coherence tomography (OCT) of the macula and optic discs, and fundus fluorescein angiography, showed no neovascularisation or ischemia. MRI of the head and orbits excluded deeper vascular anomalies. Anterior segment imaging confirmed a right eye vascular tuft at 11 o'clock on the pupillary margin, with potential other vascular tufts or pigmentary clumps around the pupil margin. As for the left eye, two vascular tufts were noted at 11 o'clock and 5 o'clock. Iris fluorescein angiography revealed bilateral hyperfluorescent lesions showing leakage (Figure 2). Anterior segment OCT demonstrated nodular thickening corresponding to angiographic findings (Figure 3).

These findings confirmed bilateral iris microhaemangiomas. The patient was managed conservatively with regular follow-up over six months, maintaining 6/6 visual acuity in both eyes, normal IOPs, and no recurrence of bleeding.

## Discussion

Iris microhaemangiomas account for only 5% of iris vascular tumours, and vascular tumours themselves comprise just 2% of all iris tumours [3]. Although typically asymptomatic, the most common complication is spontaneous hyphema. One reported case involved complete spontaneous hyphema secondary to iris microhaemangioma with an IOP of 72 mmHg, posing a significant risk of permanent vision loss if not promptly managed [4].

Due to the rarity of these lesions, there is limited consensus on optimal management. Most cases resolve with conservative treatment, including bed rest, IOP-lowering agents such as acetazolamide (contraindicated in sickle cell disease) or topical drops, mydriatics, and topical steroids to control inflammation. In cases of recurrent hyphema, laser photocoagulation may be considered, while iridectomy is reserved for cases where malignancy is suspected or when repeat laser is ineffective [1].

In this case, intravenous tranexamic acid achieved haemostasis within 30 minutes without adverse effects, suggesting it may be a quick-acting and effective treatment option. Only one prior case has described the use of tranexamic acid in this context, where 500 mg orally halted bleeding at 1 hour, also without adverse effect or re-bleeding [5].

A single intravenous dose of tranexamic acid is generally well tolerated, with only mild adverse effects such as nausea, vomiting, diarrhoea, headache, or injection-site discomfort. It is typically administered by slow injection or infusion; in this case, a 1 g dose diluted in 100 ml of 0.9% saline was infused over 10 minutes to prevent transient hypotension associated with rapid administration [6]. Serious but uncommon adverse events, including seizures and drug accumulation in patients with impaired renal function, are usually linked to high or repeated doses; however, at the dosage used in this case, there is no evidence of increased seizure risk or accumulation in renal impairment [7]. Evidence from meta-analyses also shows that intravenous tranexamic acid, irrespective of dose, is not associated with an increased thromboembolic risk, even in patients with prior thromboembolic events [8,9].

Intravenous administration achieves therapeutic levels within minutes, hence offering immediate effect, while oral may take hours to reach effective concentrations and effect [10,11]. Hence, in suitable cases where conservative management has been ineffective, intravenous tranexamic acid could be a low-risk management option that can quickly achieve haemostasis. This would enable a more timely and safe discharge than just conservation measures alone or using oral tranexamic acid.

Topical tranexamic acid has also shown similar efficacy to oral formulations in managing traumatic hyphema, though further studies are warranted to assess its role in traumatic and spontaneous cases [12]. This, however, could potentially be a management option in the future.

## Conclusions

Intravenous tranexamic acid could be considered as a low-risk, and potentially effective option in the management of spontaneous hyphema secondary to iris microhaemangiomas in patients who have not responded to conservative measures such as bed rest or ocular compression. It cannot be ruled out that this bleed resolved spontaneously; however, given the timing of haemostasis and the pharmacology of tranexamic acid, it is likely to have at least contributed. Further studies are needed to evaluate the efficacy of tranexamic acid in such cases, including comparisons of different routes of administration and dosing regimens.
